# Electrospun Nanofiber and Cryogel of Polyvinyl Alcohol Transdermal Patch Containing Diclofenac Sodium: Preparation, Characterization and In Vitro Release Studies

**DOI:** 10.3390/pharmaceutics13111900

**Published:** 2021-11-09

**Authors:** Shafizah Sa’adon, Mohamed Nainar Mohamed Ansari, Saiful Izwan Abd Razak, Abdul Halim Mohd Yusof, Ahmad Athif Mohd Faudzi, Suresh Sagadevan, Nadirul Hasraf Mat Nayan, Joseph Sahaya Anand, Khairul Anuar Mat Amin

**Affiliations:** 1 BioInspired Device and Tissue Engineering Research Group, Faculty of Engineering, School of Biomedical Engineering and Health Sciences, Universiti Teknologi Malaysia, Skudai 81300, Johor, Malaysia; shafizahsaadon@gmail.com; 2 Institute of Power Engineering, Universiti Tenaga Nasional, Kajang 43000, Selangor, Malaysia; 3 Faculty of Engineering, School of Chemical and Energy Engineering, Universiti Teknologi Malaysia, Skudai 81300, Johor, Malaysia; halimy@cheme.utm.my.edu.my; 4 Centre for Artificial Intelligence and Robotics, Universiti Teknologi Malaysia, Kuala Lumpur 54100, Malaysia; athif@utm.my; 5 Nanotechnology & Catalysis Research Centre, University of Malaya, Kuala Lumpur 50603, Malaysia; drsureshnano@gmail.com; 6 Faculty of Engineering Technology, Universiti Tun Hussein Onn Malaysia, Batu Pahat 86400, Johor, Malaysia; nadirul@uthm.edu.my; 7 Sustainable and Responsive Manufacturing Group, Faculty of Mechanical and Manufacturing Engineering Technology, Universiti Teknikal Malaysia Melaka, Hang Tuah Jaya, Malacca 76100, Malacca, Malaysia; anand@utem.edu.my; 8 Faculty of Science and Marine Environment, Universiti Malaysia Terengganu, Kuala Nerus 21030, Terengganu, Malaysia; kerol@umt.edu.my

**Keywords:** electrospinning, nanofibers, cryogelation, diclofenac sodium, polyvinyl alcohol, dual layer, transdermal drug delivery, in vitro release, Franz diffusion

## Abstract

Transdermal drug delivery systems (TDDS) have drawn more interest from pharmaceutical scientists because they could provide steady blood levels and prevent the first-pass metabolism over a longer period. Polyvinyl alcohol (PVA) has been widely used in this application due to its biocompatibility, non-toxicity, nanofiber and hydrogel-forming ability. Despite those benefits, their morphology would easily be destroyed by continuous water absorption and contribute to burst drug release due to its hydrophilicity. The aim of this study was to prepare the diclofenac sodium (DS)-medicated dual layer PVA patch using a combination of electrospinning and cryogelation (freeze–thaw) methods to improve the physicochemical properties and drug compatibility and investigate the release of the DS-medicated dual layer PVA patch. Morphological observations using scanning electron microscopy (SEM) verified the polymer−polymer interaction between both layers, whereas Fourier transform infrared (FTIR) spectroscopy has demonstrated the compatibility of DS in PVA matrix up to 2% *w*/*v* of PVA volume. The DS loads were found amorphously distributed efficaciously in PVA matrix as no visible spectra of DS–PVA interaction were detected. The DS-medicated dual layer PVA patch with a thicker nanofiber layer (3-milliliter running volume), three freeze–thaw cycles and 2% DS loading labeled as 2%DL_B_3C show the lowest swelling capacity (18.47%). The in vitro assessment using Franz diffusion cells showed that the 2%DL_B_3C indicates a better sustained release of DS, with 53.26% of the DS being released after 12 h. The 2%DL_B_3C owned a flux (*J*_ss_) of 0.256 mg/cm^2^/h and a permeability coefficient (K_p_) value of 0.020 cm/h. Thus, the results demonstrate that DS-medicated dual layer PVA patches prepared via a combination of electrospinning and cryogelation are capable of releasing drugs for up to 24 h and can serve as a drug reservoir in the skin, thereby extending the pharmacologic effects of DS.

## 1. Introduction

A transdermal patch or skin patch is a medicated patch intended to administer a particular dosage of drugs through the skin into the bloodstream and may be seen as the desired alternative to oral drug delivery [1,2]. Since they provide many benefits over other conventional analgesic administration approaches, transdermal drug delivery systems (TDDSs) are intriguing to oral drug delivery technologies [3]. A TDDS is a drug delivery technique that uses the skin to achieve a systemic or local effect. After injection and oral medication, it was classified as a third-generation medicinal preparation. Several technologies have been developed to enable rate control of drug release and transdermal permeation [4]. The primary purpose of TDDSs is to efficiently deliver drug molecules at the recommended therapeutic level to the target cell, tissue, or organ for a given period at a required dosage and rate, as well as to distribute associated drugs using suitable drug carriers to provide continuous release systems [5]. In addition, a TDDS has stable plasma levels, easy opioid monitoring and a reduced risk of overdosing [6]. Furthermore, it eliminates the effects of the gastrointestinal environment on medication effectiveness, such as enzymatic action, pH and food–drug interferences [7].

Polyvinyl alcohol (PVA) has recently been an efficient transdermal transport carrier [8,9,10,11]. PVA is a nontoxic, biocompatible and biodegradable synthetic polymer with good hydrogel-forming and electrospinnability that has been extensively used in biomedical applications [12,13,14,15]. Natural polymers such as chitosan, gelatin, agarose, alginate and starch have been used in biomedical, water treatment and food packaging [16,17,18,19]. Contrasting natural sources, PVA provides flexibility and mechanical stability to the traditional scaffolds prepared from natural polymers [20].

Nonsteroidal anti-inflammatory drugs (NSAIDs) are medications that relieve discomfort, fever, blood clotting and inflammation when used at high doses [21]. Oral NSAIDs cause various side effects, the majority of which are gastrointestinal (GI) in nature and range from stomach pain to gastric bleeding and/or ulceration of the bowel wall, liver damage and hypersensitivity reaction [22]. NSAIDs (e.g., diclofenac, ketoprofen and naproxen) are used to treat inflammation by inhibiting the production of prostaglandins in the body. Inhibiting the inducible cyclooxygenase (COX-2) enzyme prevents the development of ‘harmful’ prostaglandins [21,23]. Diclofenac sodium (DS) is a well-known anti-inflammatory nonsteroidal anti-inflammatory agent (NSAID) that has long been used to treat musculoskeletal and inflammatory conditions. It is used to treat arthritis-related swelling and inflammation by reducing rheumatic pain and delaying the immediate damage to nonarticular soft tissue caused by the disease [24]. DS is a carboxylic acid derivative and acts as a selective cyclooxygenase (COX-2) inhibitor that binds to prostaglandin receptors. One of the primary advantages of diclofenac derivatives over other traditional NSAIDs is that they inhibit the cyclooxygenase-2 (COX-2) enzyme with a higher potency than COX-1 [25]. The effectiveness of DS (even at a low dose of 100 mg/day) has been seen in studies comparable to that of other newer analgesics used to treat arthritis. Apart from its efficacy, DS has some drawbacks, including a fast metabolism owing to its short half-life (1–2 h), high protein binding and a very high pre-systemic metabolism. GI irritation, peptic ulceration and GI bleeding are also signs of diclofenac use. The low water solubility of DS causes slow dissolution and bioavailability [26].

Electrospinning is a method of creating micro/nanofibrous mats out of polymeric solutions or melts (natural and synthetic). The most straightforward and low-cost electrospinning method, which is most often used in the experimental setups mentioned in the literature, consists of a nozzle, a grounded collector, a high-voltage power supply and a mechanism that regulates the flow rate of the spinning material—either a polymer solution or polymer melt [27,28]. According to Vass et al. [29] and Zhang et al. [30], one of the apparent advantages of electrospinning over traditional film-casting techniques is the highly porous nature of the electrospun nanofiber membrane, which has a larger surface area, which could enable drug molecules to diffuse more efficiently from the matrix to the skin. However, Li et al. [31] discovered that the drug would rapidly dissolve in water in a burst manner from electrospun PVA nanofibrous matrices (caffeine to 100% and riboflavin to 40% within 60 s), which increased the risk of the body being abruptly overloaded with the drug. Despite the excellent properties of an electrospun PVA nanofiber, it has been stated in the literature that the use of electrospun PVA nanofiber membranes in a variety of applications has frequently been avoided due to their poor mechanical properties and relatively low dimensional stability, which restrict their use in some applications [32]. Various strategies have been developed, including reinforcing nanofibers with other cellulose nanocrystals, syntactic foam and well-aligned cellulose [33,34,35]. These processes, however, have been complicated and expensive.

The development of the PVA cryogel has generated significant interest as a drug carrier through freezing–thawing techniques (cryogelation), which were used to avoid the chemical crosslinking of PVA and the potential toxicity and leaching problems [36]. In agreement with Gupta et al.’s [37] finding, PVA cryogels prepared by freeze–thaw cycles are a logical alternative for this application because they exhibit a high degree of durability, a rubbery elastic nature, non-toxic, mechanically robust cryogels by hydrogen bond induced crosslinking and are readily accepted by the body.

To overcome these limitations, a cost-effective dual layer PVA patch was developed using a combination of electrospinning and cryogelation of PVA, which optimized both the physicochemical and therapeutic efficacy of transdermal drug release. Thus, DS was included in this study as a drug model. The efficacy of the DS dual layer PVA patches was determined by varying the thickness of the electrospun PVA nanofibers and the drug loading percentage. The prepared patches were characterized using a variety of physicochemical parameters prior to in vitro drug release assessment.

## 2. Materials and Methods

### 2.1. Materials

Poly (vinyl alcohol) (PVA, molecular weight ~89,000–98,000 g/mol, 99%+ hydrolyzed) was purchased from Sigma-Aldrich (St. Louis, MO, USA), with distilled water as a solvent. Diclofenac sodium (DS) ≥ 99.5% USP, molecular weight 318.13 g/mol, from the Emory laboratory. Ethanol of approximately 95%, AR Grade, was purchased from QRëC^TM^ (Chonburi, Thailand). Phosphate buffer saline solutions, PBS, 1X, pH 7.4) purchased from Gibco™ LS10010049 (Waltham, MA, USA). Whatman^TM^ Cellulose Nitrate Membrane (Marlborough, MA, USA). All other reagents were of analytical grade and used without further purification.

### 2.2. Methods

#### 2.2.1. Production of the Electrospun PVA Nanofiber Membrane

A weighed amount of PVA powder was dissolved in distilled water at 80 °C for 2 h to prepare a PVA solution at a fixed concentration of 10% *w*/*v*. After that, the solution was cooled down to room temperature (25 °C). Electrospinning of the freshly prepared PVA solutions was carried out by connecting the emitting electrode of positive polarity from a high voltage power supply model ES30PN/M692 by Gamma High Voltage Research (Ormond Beach, FL, USA) to the solutions contained in a standard 5-milliliter syringe. The open end of this was attached to a blunt gauge-23 stainless steel needle (outer diameter = 0.91 mm), used as the nozzle, and the collection plate was laminated with aluminum foil (dimension = 15 cm × 15 cm), used as the fiber collection device. A fixed electrical potential of 20 kV was applied across a fixed distance of 15 cm between the tip of the nozzle and the outer surface of the collector plate (i.e., the electrostatic field strength of (20 kV/15 cm). The feed rate of the solutions was controlled to about 1 mL/h utilizing a syringe pump.

#### 2.2.2. Preparation of the PVA Cryogel Containing Diclofenac Sodium (DS)

The aqueous PVA solution was prepared prior to the cryogelation process as described in Section 2.2.1. The PVA was completely dissolved, and the clear solutions obtained were gradually cooled to room temperature. As for DS solution, different mass % of DS (1.0, 1.5 and 2.0 % *w*/*v*) was initially dissolved in 5 mL of ethanol in a separate beaker, then slowly transferred into freshly prepared PVA solutions and heated to prevent re-precipitation.

#### 2.2.3. Preparation of the Dual Layer PVA Patches

The aqueous PVA solutions (from Section 2.2.2) were then spread on top of the electrospun PVA nanofibers membrane, which had been placed in a custom-designed mold with the dimensions of 8 cm × 8 cm × 1.5 cm (W × L × H). Cryogelation of a dual layer PVA patch was achieved by repeatedly freezing–thawing the PVA aqueous solutions (24 h freezing at −20 °C followed by a 2-hour thawing process at room temperature). The following Table 1 summarizes the formulation of unmedicated and diclofenac sodium (DS)-medicated dual layer PVA patches:

### 2.3. Morphological Observation

#### Morphology of Dual Layer PVA Patches (Unmedicated and DS-Medicated)

The prepared patches’ cross-sections were analyzed morphologically using a scanning electron microscope (SEM) model JEOL-JSM6380LA (Tokyo, Japan) operating at 15 kV with a 30-meter magnifier under high vacuum. The freeze-dried samples were cut to tiny dimensions and subsequently sent to an Auto Fine Coater Machine to have a thin film of gold sputtered on their surface at a plasma current of 30 mA and a chamber pressure of 2 Pa. The coating aims to ensure the electrical conductivity of the insulating freeze-dried cryogel samples during high-resolution electron imaging applications.

### 2.4. Physicochemical Characterization

#### 2.4.1. Fourier Transform Infrared Spectroscopy (FTIR) of the Dual Layer PVA Patch

FTIR is used to investigate the interaction between the drug model and polymer of the dual layer PVA patch. Prepared dual layer PVA patches were cut into a small cube (10 mm × 10 mm × 10 mm) and placed at the FTIR sample holder. FTIR spectra were recorded in the range of 600 to 4000 cm^−1^ collecting 35 scans with a 4 cm^−1^ resolution in the transmittance mode.

#### 2.4.2. Swelling Capacity of the Dual Layer PVA Patches

The dry dual layer PVA cryogel was soaked in PBS (pH 7.4) at various time intervals before an equilibrium state of absorption was reached. After filtering out excess surface water with filter paper, the swollen gel’s weight was determined at different time intervals. This process was replicated until no further weight gain was observed. Equation (1) can be used to calculate the equilibrium swelling ratio (ESR) as a function of time.

(1) $$\mathrm{ESR}\left(\%\right)=\frac{W_{t}{-W}_{i}}{W_{i}}\times 100,$$

where W_t_ = the weight of the dual layer PVA patch after swelling at time interval, and W_i_ = the initial weight of the dual layer PVA patch.

### 2.5. Preparation of Standard Calibration Curve of Diclofenac Sodium (DS)

For the estimation of DS, a simple, precise, accurate and cost-effective UV–visible spectrophotometric approach has been developed. Calibration is an essential step in analytical methods, and it is necessary to assess the results obtained. The standard solution for the DS was prepared using PBS solution (pH 7.4) from the stock solution at 10, 20, 30, 40 and 50g/mL concentrations. The absorbance of pure DS solutions was estimated at 276 nm (λmax), and a calibration curve was plotted between the concentration of DS (g/mL) on the x-axis and the absorbance on the y-axis.

### 2.6. In Vitro Release Assessment of Diclofenac Sodium (DS) Dual Layer PVA Patches Using Franz Diffusion Cell

**Figure 1:**
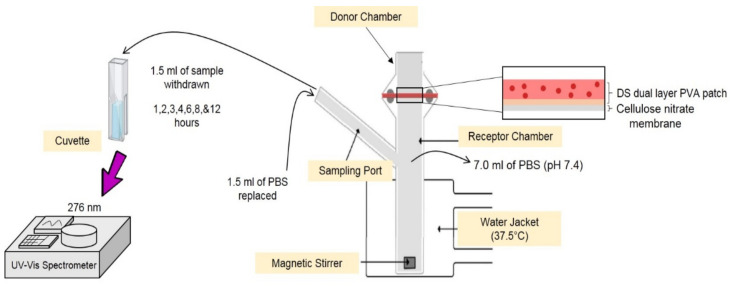
Franz diffusion cell set up for diffusion studies.

The release experiments were conducted using a Franz diffusion cell (as refer to Figure 1). A cellulose nitrate (skin-mimicking membrane) was placed between the donor and receptor chambers of the Franz diffusion cell. A water jacket attached to a water bath was used to preserve the temperature of the receptor solution at 37 °C. Each patch was cut into a 4 cm^2^ and placed on top of a cellulose nitrate membrane, then mounted on a receptor chamber containing 7.0 mL of PBS solution (pH 7.4). In vitro DS release was monitored using a Franz diffusion cell for 1, 2, 3, 4, 6, 8 and 12 h.

The withdrawn solution (1.5 mL) in the receptor chamber was then replaced with the same volume of PBS solution (pH 7.4) to keep the sinking volume consistent throughout the process. The concentration of DS in the sample solution was determined using a UV–visible spectrophotometric preset to 276 nm. The cumulative release of DS from dual layer PVA patches was calculated using the data collected.

### 2.7. In Vitro Drug Release from Diclofenac Sodium (DS)-Medicated Dual Layer PVA

#### 2.7.1. Calculation of Cumulative Amount of Drug Release

The cumulative amount of drug released (Equation (2)) was calculated at each interval and the cumulative release per area was plotted versus time.

(2) $$Q_{t}{=V}_{r}{\times C}_{r}\overset{t=1}{\underset{i=0}{\sum }}V_{s}{\times C}_{I}$$

Q_t_ = Cumulative drug release at t interval

V_t_ = Volume receptor medium

C_r_ = Concentration of drug in receiving chamber at each sampling time

V_s_ = Sampling volume

C_i_ = Concentration of drug for i^th^ sample

#### 2.7.2. Data Analysis of the In Vitro Permeation of DS-Medicated Dual Layer PVA Patch

The steady-state fluxes, (*J_ss_*), (mg/cm^2^/h) (the slope of the linear portion of the permeation curve), expressed as the mass of drug passing across patch over time, were calculated using the following Equation (3):

(3) $${\mathrm{Flux},J}_{\mathrm{ss}}=\frac{∆\mathrm{Qt}}{∆t\cdot S}$$

Apparent permeability coefficients (K_p_, cm/h) were calculated according to the following Equation (4):

(4) $${\mathrm{Permeability}\;\mathrm{coefficients},\;K}_{p}{=J}_{\mathrm{ss}}\cdot {\;C}_{d}$$

where K_p_ was the permeability coefficient, *J_ss_* was the flux calculated at a steady-state, and C_d_ represented the DS concentration that remained constant in the vehicle, and it was assumed that under sink conditions, the DS concentration in the receiver compartment was negligible. Lag time (tLag) was determined from the X-intercept of the regression line [38].

## 3. Results and Discussions

### 3.1. Preparation of Unmedicated and Diclofenac Sodium (DS)-Medicated Dual Layer PVA Patches

**Figure 2:**
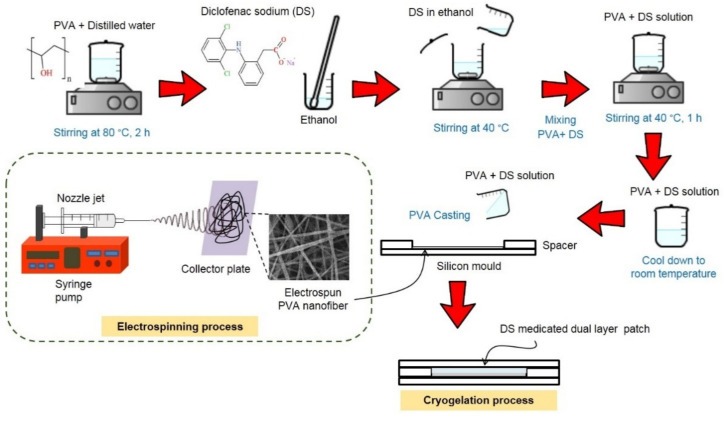
Preparation process of DS-medicated dual layer PVA patches.

The unmedicated and DS-medicated dual layer PVA patches were successfully prepared using a combination of the electrospun nanofiber membrane and cryogel of PVA. The preparation and layering process of the electrospun nanofiber membrane and cryogel of PVA are depicted in Figure 2. As the cryogelation (freezing) process occurred, the PVA solutions induce the amorphous region to form ice crystals, enabling the polymer chains to assemble into small, structured crystallite regions [39].

### 3.2. Morphological Structures of the Dual Layer PVA Patches

According to Hassan and Peppas [40], the gelation process of PVA results in the formation of PVA polymeric chains that are linked together by hydroxyl (-OH) bonds and separated by weaker van der Waals forces. In addition, due to the effect of strong cross-linked PVA, the polymer network constricting throughout the freezing and thawing processes produced the non-porous structures [41].

**Figure 3:**
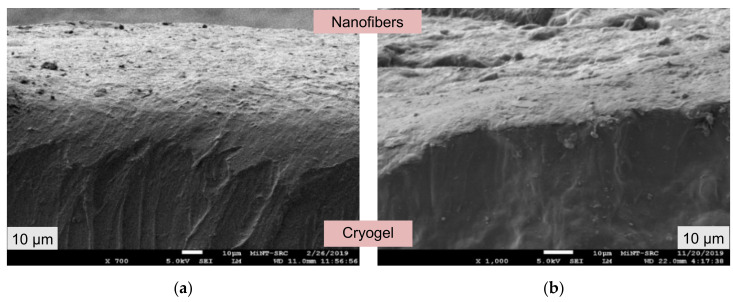
Cross-section of prepared dual layer P VA patches, (**a**) DL_A_3C and (**b**) DL_B_3C.

As can be seen, Figure 3a,b show a cross-sectional observation of the dual layer of the PVA patches (the cryogel and electrospun PVA nanofiber membrane) after completing the preparation process. Theoretically, due to the hydrophilicity of PVA, the dual layer PVA patches demonstrated good polymer interaction between electrospun nanofiber membrane and cryogel, as shown in Figure 3. It is also worth noting that the nanofiber membrane in Figure 3b is slightly thicker than the nanofiber membrane in Figure 3a.

### 3.3. Compatibility of Diclofenac Sodium (DS) in the Dual layer PVA Patches

**Figure 4:**
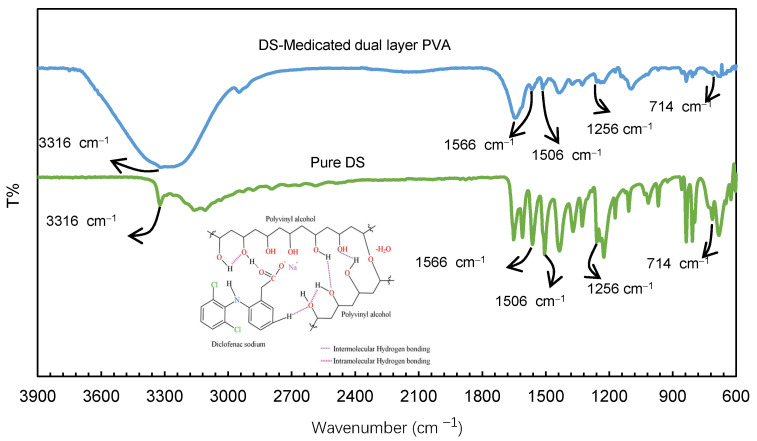
FTIR spectra of diclofenac sodium (DS) compatibility in dual layer PVA patches.

The presence of DS in the dual layer PVA patch was confirmed using FTIR spectroscopy. The FTIR spectra of pure DS and DS-medicated dual layer PVA patch are shown in Figure 4.

According to Baghel et al. [42], active pharmaceutical ingredients (APIs) can exist in either a crystalline or amorphous state. Based on the interpretation, the FTIR spectra of the DS-medicated dual layer PVA patch showed the characteristic peaks of the pure DS. This indicates the amorphous presence of DS within the dual layer patch, rather than being covalently bonded to the PVA polymer network. As can be seen in Figure 4, the spectra revealed a distinct peak at 3316 cm^−1^ due to the N−H stretching of the secondary amine. Additionally, peaks were observed at 1566 and 1506 cm^−1^, respectively, due to the carboxyl group (−C=O stretch) and the aromatic compound (C=C stretch) [43]. The FTIR spectrum at 1256 cm^−1^ was generated by aromatic amine C−N stretching [44,45], and C−Cl stretching vibrations, on the other hand, exhibit peaks in the range of 650−780 cm^−1^ [46].

### 3.4. Effect of Nanofiber Thickness and Drug Loading Percentages on Swelling Capacity of the Dual Layer PVA Patches

**Figure 5:**
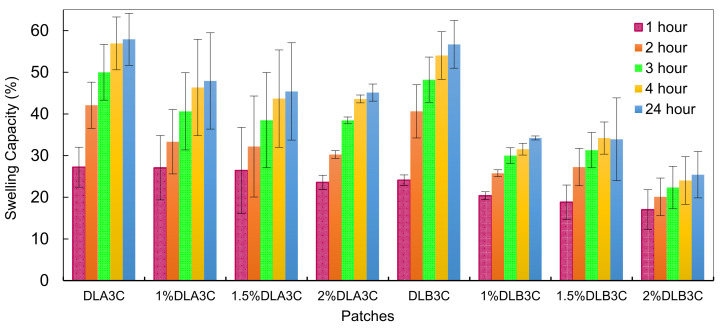
Unmedicated and DS-medicated DL_A_3C and DL_B_3C swelling capacity as a function of immersion time and DS loading percentages.

The amount of water absorbed by the drug and its efficacy for biomedical applications are significant considerations in determining its compatibility. The utilization of DS as a drug model and the effect of DS loading percentages on the swelling property of the dual layer PVA patch is also a critical factor in this study. Figure 5 depicts the swelling capacity (%) of the DL_A_3C and DL_B_3C. From the results obtained, both parameters are closely related to the swelling behavior of the prepared dual layer PVA patches.

The swelling capacity (%) of dual layer PVA patches with various nanofiber thickness and DS loading percentage is compared to attain a comparative effect. As can be seen in Figure 5, the swelling capacity (%) of the DS-medicated DL_A_3C increased up to 47.91% and then decreased marginally to 45.13 % after 24 h, while the swelling capacity (%) of the DS-medicated DL_B_3C decreased from 30.11 to 18.47%. In contrast, the unmedicated dual layer PVA patches show a noticeably higher percentage of swelling capacity.

Furthermore, these findings are in accordance with an increase in the crystallinity of the dual layer PVA patch due to the addition of a thicker nanofiber to the PVA cryogel during the combined process. The increased amount of the hydroxyl group (-OH) in polymer chains is potentially due to the increased intermolecular or intramolecular interactions between the two layers (nanofiber and cryogel), resulting in an increase in patch crystallinity after the combine process is completed.

To understand the effect of drug saturation on swelling behavior, the swelling pattern of DS-medicated dual layer PVA patches from 1 to 2 (% *w*/*v*) of the DS loading was observed. From the results obtained, it can be concluded that the water absorption capacity for both thicknesses decreased constantly with increasing percentages of the DS loading.

Overall, the decreased swelling capacity was affected by incorporating DS molecular structures into PVA polymer chains, resulting in narrow cryogel pores [47], leading to a reduced swelling capacity of the DS-medicated dual layer PVA patch.

### 3.5. Morphological Structures of Electrospun PVA Nanofiber Layer Side after Diffusion Process

**Figure 6:**
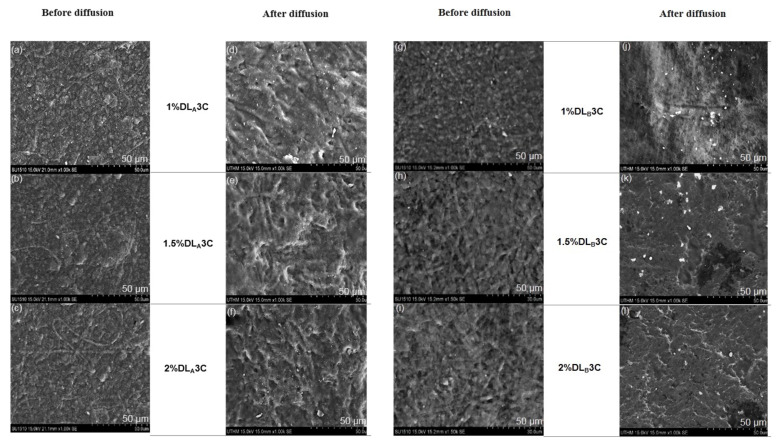
Morphological structures of the dual layer PVA patches (nanofiber side) before and after diffusion process: (**a**−**f**) DS-medicated DL_A_3C and (**g**−**l**) DS-medicated DL_B_3C.

To assess the stability of the dual layer PVA patch after drug release, Figure 6a,b depict the morphological structure (nanofiber layer side) after a 12-hour diffusion process. Each patch was removed after the completion of the process and sent to the SEM for observations.

The nanofiber layer side of the DS-medicated DL_A_3C and DL_B_3C has changed substantially after 12 h of diffusion. The continual adsorption and dissolution of the electrospun PVA nanofiber layer during the process could have been inducing the expansion of PVA nanofibers. Furthermore, after a 12-hour diffusion process, all the formulations for dual layer PVA patches demonstrate drug–polymer stability. Continued studies on the in vitro release of the DS-medicated dual layer PVA patch can substantiate this statement.

### 3.6. In Vitro Evaluation of Diclofenac Sodium (DS)-Medicated Dual Layer PVA Patches Release

**Figure 7:**
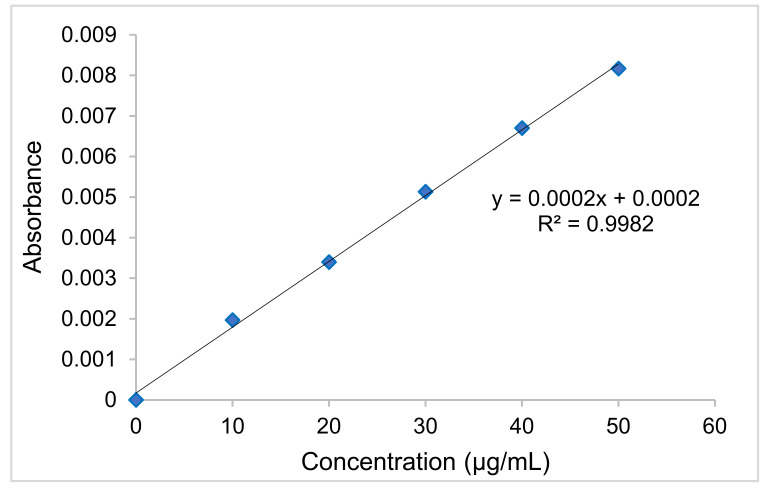
Standard calibration curve of diclofenac sodium (DS).

In biomedical drug delivery research involving transdermal delivery, the drug release parameter is crucial. With the nanofiber side facing the cellulose nitrate membrane, each formulation of DS-medicated DL_A_3C and DL_B_3C was mounted on the top of the receptor compartment of a Franz diffusion cell containing 7.0 mL of PBS solution. Subsequently, the DS release profiles were evaluated at various time intervals to estimate the drug’s release rate within 12 h. Figure 7 depicts the standard calibration curve for DS to determine the percentage of cumulative drug release (% CDR).

**Figure 8:**
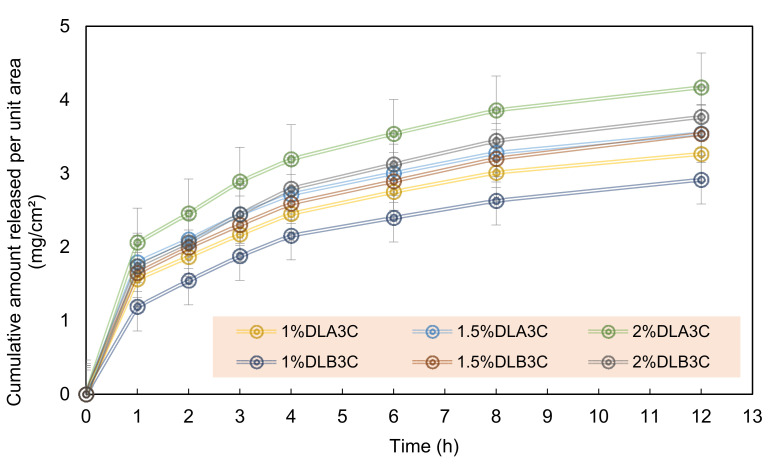
Cumulative amount of DS release per unit area (mg/cm^2^) for DS-medicated DL_A_3C and DL_B_3C. Each data point represents mean ± SD of three measurements.

The cumulative amount of drug released per unit area (mg/cm^2^) over time (h) was plotted in Figure 8 for DS-medicated DL_A_3C and DL_B_3C.

Taking the 1.0 (% *w*/*v*) concentration into consideration, the initial DS drug released for 1% DL_B_3C is significantly lower at 1.19 mg/cm^2^ (33.65% total drug per unit area) compared to 1% DL_A_3C at 1.56 mg/cm^2^ (44.23%) for the first 1 h of diffusion. Nonetheless, the initial release in this study was lower than the initial release of DS via an electrospun nanofiber, which reached up to 49%, as reported by El-Newehy et al. [48]. The higher %CDR of DL_A_3C was associated with the high swelling capacity of the patches, resulting in a more relatively rapid release of the DS from the dual layer PVA patches into the medium (refer to Section 3.4).

After 12 h of diffusion, the amount of DS released per unit area is 3.26 (92.31%), 3.54 (66.60%) and 3.77 mg/cm^2^ (58.94%), respectively, for 1% DL_A_3C, 1.5% DL_A_3C and 2% DL_A_3C, whereas for DL_B_3C, the amount of DS released is 2.91 (82.40%), 3.54 (66.47%) and 3.77 mg/cm^2^ (53.26%), respectively, for 1.0, 1.5 and 2.0 (% *w*/*v*). The collected data indicate that the amount of drug released decreases as the nanofiber in the dual layer PVA patch thickens. With an increasing DS loading, a similar pattern can be observed. As a result of drug saturation in the PVA matrix, the % CDR decreased as the DS percentage loading increased (from 1.0 to 2.0% *w*/*v*).

### 3.7. In Vitro Permeation Studies of the Diclofenac Sodium (DS)-Medicated Dual Layer PVA Patches through Cellulose Nitrate Membrane

**Figure 9:**
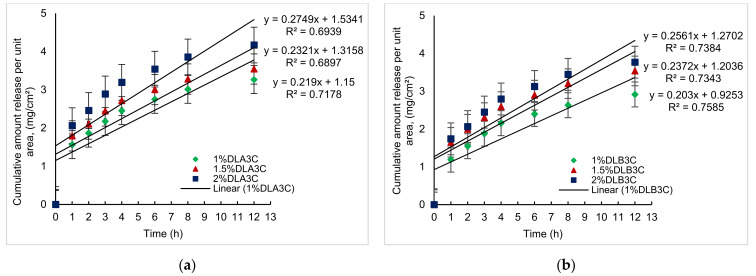
Plotted graph for in vitro permeation profiles of DS medicated: (**a**) DL_A_3C (1 to 2 % *w*/*v*); (**b**) DL_B_3C (1 to 2% *w*/*v*).

In vitro studies using excised skin tissue are widely used to perform transdermal diffusion studies. However, experiments involving excised human skin often exhibit significant intra- and inter-subject variation, are costly and time-consuming and raise ethical concerns [49,50]. Alternatively, semi-synthetic cellulose nitrate membranes mimicking skin have been selected as in vitro model membranes. This study aimed to identify the quantity of DS that efficiently permeates the cellulose nitrate membrane using various formulations. The in vitro permeation profile of the DS-medicated dual layer PVA patch was generated by plotting the cumulative amount of drug permeated per unit area (mg/cm^2^) over time (h), as shown in Figure 9.

The steady-state flux, *J_ss_* (mg/cm^2^/h) of DS-medicated dual layer PVA patches, was determined as the slope of the linear regression line. *J_ss_* is described as the rate at which a substance diffuses or transports through a permeable membrane. Based on in vitro drug release data, as stated in Section 3.6, in vitro permeable experiments were conducted. Table 2 summarizes the steady-state flux, *J_ss_*, linear regression, R^2^, permeability coefficient, K_p_, and lag time (tLag) obtained in this analysis.

As shown in Table 2, the DL_B_3C exhibits a lower flux value of 0.203 to 0.256 mg/cm^2^/h than the DL_A_3C, which exhibits a range of 0.219 to 0.275 mg/cm^2^/h. Furthermore, as the DS loading increased from 1.0 to 2.0 (% *w*/*v*), the flux value also increased. In theory, lag time (tLag) is the time taken for the drug to be released from the reservoir and become permeable to reach a constant release rate [51,52,53,54]. As referring to Table 2, the lag time for DS-medicated DL_A_3C to attain its steady-state was observed to be slight longer than DL_B_3C as the nanofiber thickness and DS loading increased, whereas the permeability coefficient (K_p_) of DL_A_3C was marginally higher (0.035 to 0.022 cm/h) than that of DL_B_3C (0.032 to 0.020 cm/h) as the nanofiber thickness and DS loading percentage increased. According to Kumar and Kotain [55], the permeability coefficient, K_p_, represents the velocity of drug passage through the skin or membrane. In this case, the decreased drug permeation (K_p_ value) may be attributed to the drug’s mobility being restricted due to greater intermolecular bonding between the -OH group in PVA chains and the drug molecules.

In summary, the different nanofiber thickness and DS loading percentages affect the permeability of the drug passing through the cellulose nitrate membrane. Based on these permeability studies, it is apparent that drug permeation is related to drug release, as discussed in Section 3.6. The DL_A_3C and DL_B_3C were found to be optimal for drug release due to an appropriate lower flux (0.35 mg/cm^2^/h) after 12 h of permeation. The higher the flux is, the quicker the pain relief is, while a slower flux can result in a delayed release of the drugs, which can be applied once, and the therapeutic effect can last up to 24 h. Nonetheless, by adjusting the formulations, the amount of the drug released can be varied.

### 3.8. Proposed Diclofenac Sodium (DS)-Medicated Dual Layer PVA Patch for Commercial Use

**Figure 10:**
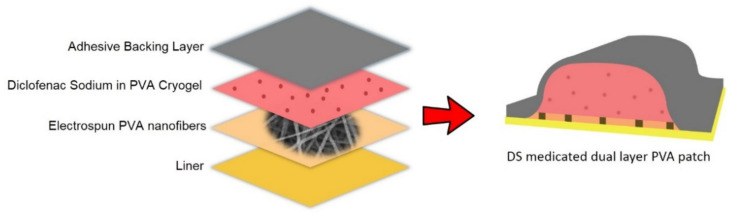
Components of proposed DS-Medicated dual layer PVA patch.

This study combined nanofiber mats and PVA cryogel to create a patch that functioned as both a drug reservoir and a release membrane, which is the primary drug transport mechanism. As shown in Figure 10, a DS-medicated dual layer PVA patch can be constructed by combining several components, including a reservoir (PVA containing DS), an adhesive backing layer, PVA nanofiber mats and a liner.

**Figure 11:**
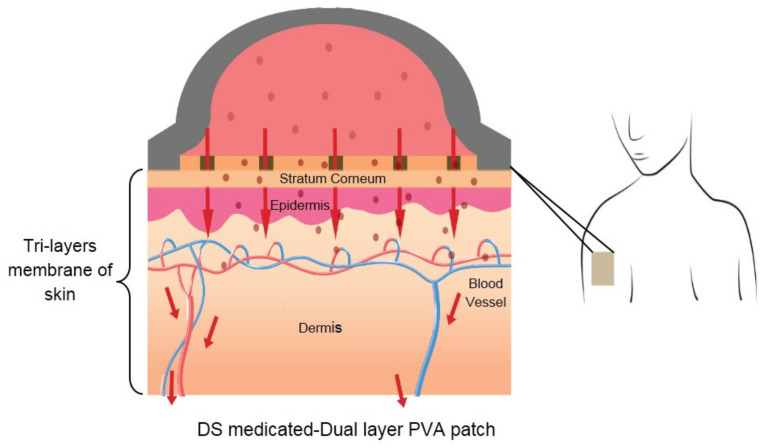
Transdermal mechanism of DS-medicated dual layer PVA patch through skin.

Figure 11 depicts a DS-medicated dual layer PVA transdermal delivery mechanism after applying the patch to the skin surface. Once the drug is released from the patch, it penetrates the skin’s three distinct layers: the stratum corneum (SC), the epidermis and the dermis. It is by far the most crucial barrier to drug permeation through the skin. The epidermis is a viable tissue devoid of blood vessels and is located directly under the SC. The dermis is the deepest skin layer, containing blood vessels capable of absorbing medications delivered systemically.

## 4. Conclusions

The unmedicated and DS-medicated dual layer PVA patches were successfully prepared by combining electrospinning and cryogelation (freeze–thaw) techniques. SEM verified the good layering between electrospun PVA nanofiber and PVA cryogel and the dispersion of DS in the PVA matrix. The FTIR spectrum indicated no interference between the DS and PVA, as the DS demonstrated its characteristic peak for each medicated dual layer PVA patch. The increases in both parameters resulted in a decrease in the swelling potential due to an increased cross-linking density, and the addition of DS inhibited water absorption into the dual layer PVA patch after a 24-hour immersion in PBS. The release of a DS-medicated dual layer PVA patch was investigated in vitro using Franz diffusion cells and a cellulose nitrate membrane to imitate the human skin barrier. The cumulative drug release from a DS-medicated dual layer PVA patch with the highest percentage of drug loading (2% *w*/*v*) demonstrates continuous release and the possibility of prolonged drug release up to 24 h. These studies may result in new cost-effective and environmentally friendly approaches that exclude a potentially toxic cross-linker. This novel preparation of DS-medicated dual layer PVA patch can be promising for transdermal drug delivery simultaneously, minimizes gastrointestinal side effects associated with non-steroidal anti-inflammatory drugs and satisfies the increasing demand in pharmaceutical and biomedical applications.

## Figures and Tables

**Table 1:** Formulations of unmedicated and diclofenac sodium (DS)-medicated dual layer PVA patches.

Patches	Concentration of PVA (% *w/v*)	DS Loading (% *w/v*)	No. of Freeze–Thaw Cycles
* 2 mL—volume of electrospinning			
DL_A_3C	10	-	3
1%DL_A_3C		1.0	
1.5%DL_A_3C		1.5	
2%DL_A_3C		2.0	
* 3 mL—volume of electrospinning			
DL_B_3C	10	-	3
1%DL_B_3C		1.0	
1.5%DL_B_3C		1.5	
2%DL_B_3C		2.0	

* A = 2-milliliter electrospinning running volume; B = 3-milliliter electrospinning running volume.

**Table 2:** Flux (*J*_ss_) linear regression (R^2^), permeability coefficient (K_p_) and lag time (tLag) of DS-medicated DL_A_3C and DL_B_3C.

Patches	*J*_ss_ (mg/cm^2^/h)	R^2^	K_P_ (cm/h)	(tLag)
1%DL_A_3C	0.219	0.718	0.035	1.150
1.5%DL_A_3C	0.232	0.690	0.025	1.316
2%DL_A_3C	0.275	0.694	0.022	1.534
1%DL_B_3C	0.203	0.759	0.032	0.925
1.5%DL_B_3C	0.237	0.734	0.025	1.204
2%DL_B_3C	0.256	0.738	0.020	1.270

Data are expressed by mean ± SD (n = 3).

## Data Availability

The data presented in this study are available on request from the corresponding author.
